# On Landscape Patterns in Typical Mountainous Counties Middle Reaches of the Yangtze River in China

**DOI:** 10.3390/ijerph18084000

**Published:** 2021-04-10

**Authors:** Yang Yi, Mingchang Shi, Chunjiang Liu, Hongzhang Kang, Bin Wang

**Affiliations:** 1 Shanghai Engineering Research Center of Landscaping on Challenging Urban Sites, Shanghai Academy of Landscape Architecture Science and Planning, 899 Longwu Road, Xuhui District, Shanghai 200232, China; yiyang0307@sjtu.edu.cn; 2 Beijing Engineering Research Center of Soil and Water Conservation, Beijing Forestry University, Beijing 100083, China; 3 School of Agriculture and Biology, Shanghai Jiao Tong University, Shanghai 200240, China; chjliu@sjtu.edu.cn (C.L.); kanghz@sjtu.edu.cn (H.K.); 4 Shanghai Urban Forest Ecosystem Research Station, State Forestry Administration, Shanghai 200240, China

**Keywords:** plantations, natural forests, landscape patterns, mountainous areas, Yangtze River

## Abstract

The landscape patterns of plantations (PT) are the results of human disturbances on local vegetation, and in turn, differ greatly from natural forests (NF), since the patterns strongly influence the natural circulation of material and energy. There is a need to understand the differences of landscape patterns between PT and NF, to establish a near natural afforestation strategy. This study chose three typical silvicultural counties in the middle reaches of the Yangtze River as the research areas and compared the landscape patterns of NF and PT, with other land use types (grassland, GL; cropland, CL; shrubland, SL; orchard, OR; built-up land, BUL; bare land, BL; and water bodies, WB). The results revealed that the areas of PT accounted for 7.67%, 12.05%, and 18.97% of three counties, bigger than GL, OC, BUL, BL, and WB, as one of main land use types. The landscape patterns of PT (mean patch size between 2.06 to 6.05 ha) were more fragmented than NF (mean patch size between 5.83 to 53.91 ha). NF areas increased along the relative altitude gradient, from 0 to 2500 m, while PT areas peaked from 100–1000 m. The higher the altitude, the more typical the zonal distribution of PT, the more aggregated the NF. NF had significant negative correlations with BL, BUL, CL, PT, GL, and OC, which suggest that human activities had seriously interfered with NF. Although PT as an ecological protection strategy was increasing, the landscape patterns of PT were obviously different from NF. This may affect the material energy flow in the ecological environment. The results in the present study have great implications in the other regions in China and the relevant parts of the world where natural forests were heavily disturbed.

## 1. Introduction

The patterns of natural landscape are primarily modified by anthropogenic disturbances in many regions of the world, although they are influenced by diverse factors [1,2,3]. Such patterns have profound consequences in the ecosystem and the products that they provide [4,5]. Most of landscape changes have occurred as a result of change in land use types. Land use types are defined by their anthropogenic use, such as forests, cropland, built-up land, etc. [6,7]. When land use types change, the structure and function of landscapes also change.

Using landscape indicators to define the landscape and a consideration of multiple spatial scales are the basis for understanding the complexity of space [8]. The landscape often shows different patterns at different spatial scales. An analysis from a single-scale usually ignores the details [9]. Therefore, it is necessary to select appropriate landscape indicators from multiple scales to analyze landscape patterns. Indicators can be categorized according to whether their responses are consistent or not, and there are differences in responses among different research scales [10,11]. In this context, with the same spatial scope and roughly the same proportion of land use types, the landscape features between different regions were representative and could represent the landscape patterns features of other regions with the same level of development [12,13].

Mountainous areas account for about one-quarter of the total land areas of the world. It has one-tenth of the world’s population and provides more than half of the world’s goods and services [14]. Once China sacrificed its ecological environment in pursuit of economic development, the natural forests in mountainous areas suffered a lot of damage [15,16,17]. Between 2010 and 2015, the world’s natural forest areas lost 6.5 million hectares a year, while plantations grew at a rate of more than 3 million hectares a year [18]. In this context, the “Middle Yangtze River Shelter Forest System” has been established in the Yangtze River basin [19]. Many studies have proved that the biodiversity and ecosystem services of plantations are lower than those of natural forests [20,21]. In fact, the fragmentation of forest landscape has a negative impact on biodiversity and ecosystem function [22,23], but there are few articles comparing the patterns differences between plantations and natural forests.

For this paper, three silvicultural counties, Shaoyang in Hunan Province, Shicheng in Jiangxi Province, and Zhushan in Hubei Province, were selected as the study sites. The three counties belong to typical mountainous areas and basically represented the landscape patterns of plantations and natural forests in the middle reaches of the Yangtze River. Through a multi-scale study, this study is conducted to compare the differences of landscape patterns between plantations and natural forests, and explore the impact of human activities on forests. The objectives of this study are (1) to quantify land use composition and landscape patterns in mountainous areas, (2) to examine characteristics of plantations and natural forests in mountainous areas, and (3) to discuss the relationship of forests (plantations and natural forests) distributions with other land use types. In addition, it should be noted that there were many abbreviations in this paper, thus please refer to Appendix A.

## 2. Materials and Methods

### 2.1. Study Area

**Figure 1:**
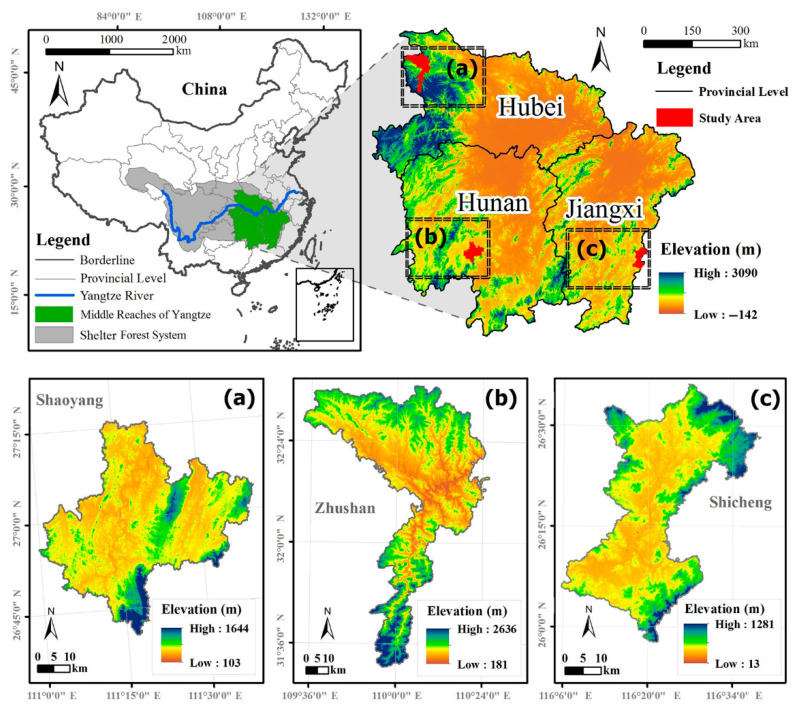
Locations of Shaoyang (SY) (**a**), Zhushan (ZS) (**b**), and Shicheng (SC) (**c**) counties in the middle reaches of the Yangtze River (MRYR).

The study areas included three typical counties, respectively, Shaoyang county (SY) in Hunan Province (110°59′–110°40′ E, 26°40′–27°06′ N), Zhushan county (ZS) in Hubei Province (109°32′–110°25′ E, 31°30′–32°37′ N), and Shicheng county (SC) in Jiangxi Province (116°05′–116°38′ E, 25°57′–26°36′ N) (Figure 1). SY belongs to a mid-subtropical monsoon humid climate area, with an annual precipitation of 1314.2 mm and annual average temperature of 17.8 °C [24,25]. ZS belongs to a subtropical continental monsoon climate, with an average annual precipitation of 905.2 mm, and median annual temperature of 15.6 °C [26]. SC belongs to a humid subtropical monsoon climate, with an average annual rainfall of 1757 mm, and annual average temperature of 18.5 °C [27,28]. The three counties all have a common feature, that is, they are located in the low hilly areas in the middle reaches of the Yangtze River, and are typical counties of planted forests. Taking these three counties as research areas, this study can preliminarily estimate the overall characteristics of landscape pattern, especially the characteristics of plantations and natural forests in the afforestation areas of the middle reaches of the Yangtze River in China.

**Figure 2:**
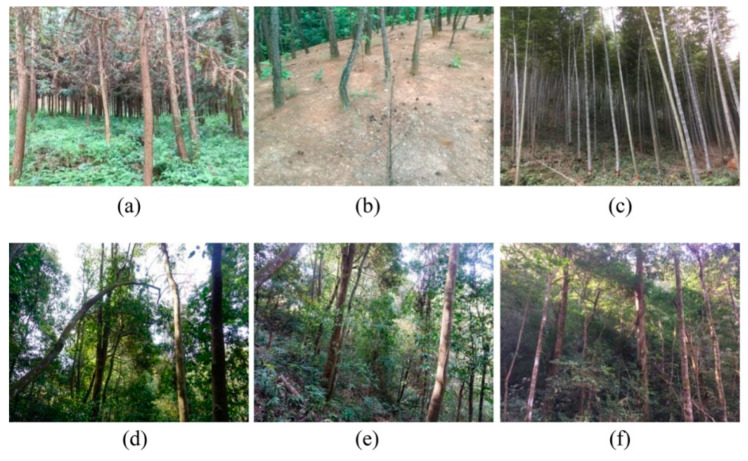
Vegetation characteristics in the study area; (**a**–**c**) plantation; (**d**–**f**) natural forests.

These three counties reside within typical mountainous areas, being far away from provincial capital cities and mid-sized cities in the Yangtze River Economic Zone, and are the key implementation areas of the “Middle Yangtze River Shelter Forest System”, which is a high proportion of plantations in the landscape. These plantations typically exist in the form of pure stands, and the understory vegetation is relatively homogeneous. The plantation species mainly include Chinese fir, Masson pine, and bamboo (Figure 2a–c). The natural forests are broad-leaved forests mainly composed of *Schima* spp., *Cinnamomum* spp., and *Quercus* spp., etc. (Figure 2d–f). The three study areas possess the dual attributes of mountainous ecosystem vulnerability and underdevelopment of regional economies.

### 2.2. Data Preprocessing

The data from 2009 and 2013 Landsat TM/OLI remote sensing images were used in this study, covering study areas in three typical counties (SY, ZS, and SC) [29,30]. The land use types in the study areas and China’s land use classification system are referred for classification. Considering the artificial forest and natural forest, the texture structure of remote sensing image is quite different. Usually plantations present regular textures. Therefore, this study applied the grayscale co-occurrence matrix to calculate eight features (energy, contrast, entropy, uniformity, mean value, variance, non-similarity, and correlation) in the image texture features and added them into the spectrum as classification features. Then, the neural network method is used to divide land use into nine categories [31]. These areas include the grass (GL), farmland (CL), shrub (SL), orchard (OC), built-up area (BUL), bare land (BL), water (WB), plantation (PT), and natural forest (NF) (Table 1). Ninety samples were randomly selected for each county (270), with high resolution image inspection Google earth (10 × 10 m) and the accuracy of interpretation, the overall accuracy, and kappa coefficient were >80%. All operations were performed using the ENVI 5.3 software.

### 2.3. Selected Pattern Metrics and Computing

Mean patch size (MPS), percentage of landscape (PLAND), interspersion juxtaposition index (IJI), largest patch index (LPI), perimeter area fractal dimension (PAFRAC), and patch cohesion index (COHESION) were selected to characterize landscape patterns [10,11]. The significance of these landscape indicators re MPS: Measures the extent of landscape fragmentation; PLAND: Measures the proportion of landscape; IJI: Reflects typical zonal distribution; LPI: Reflects dominant patch; PAFRAC: Reflects the complexity of the patch shape; and COHESION: Reflects the degree of aggregation or extension of patches (Table 2).

The following equations calculate the MPS, PLAND, IJI, PAFRAC, and COHESION. All calculations were extracted from the FRAGSTATS 4.2 software manual.

(1) $$MPS_{i}=\sum_{j=1}^{n}\frac{a_{ij}}{n_{i}}$$

where *i* is the *i*th land use type, *j* is the *j*th patch of the *i*th land use type, *a_ij_* is the *j*th patch area of the *i*th land use type, and *n_i_* is the patch number of the *i*th land use type.

(2) $$PLAND_{i}=\frac{\sum_{j=1}^{n}a_{ij}}{A}\times 100\%$$

where *a_ij_* is the area of patch *ij*, and *A* is the total landscape area, 0 < *PLAND* ≤ 100%.

(3) $$IJI=\frac{-\sum_{i=1}^{m}\sum_{k=i+1}^{m}\left[\left(\frac{e_{ik}}{E}\right)\cdot \ln\left(\frac{e_{ik}}{E}\right)\right]}{\ln\left(0.5\left[m\left(m-1\right)\right]\right)}\left(100\%\right)$$

where *e_ik_* is the total length (m) of the edge in the landscape between patch types (classes) *i* and *k*; *E* is the total length (m) of edge in the landscape, excluding outer boundary; and *m* is the number of patch types (classes) that are present in the landscape, including the landscape border.

(4) $$LPI_{i}=\frac{\max_{i=1}^{n}a_{i}}{A}\times 100\%$$

where *a_ij_* is the area of patch *i*, and *A* is the total landscape area, 0 < *LPI_i_* ≤ 100%.

(5) $$PAFRAC=\frac{\frac{2}{n_{i}\sum_{j=1}^{n}\left(\log p_{ij}-\log a_{ij}\right)-\sum_{i=1}^{n}p_{i}\sum_{i=1}^{n}a_{i}}}{n_{i}\log p_{i}{}^{2}-{\left(\sum_{i=1}^{n}\log p_{i}\right)}^{2}}$$

where $a_{i}$ is the area of patch *i* (m^2^), $p_{i}$ is the perimeter of patch *i* (m), $n_{i}$ is the number of patches in the landscape of patch type *i*, 1 ≤ *PAFRAC* ≤ 2.

(6) $$COHESION=\left[1-\frac{\sum_{i=1}^{m}\sum_{j=1}^{n}p_{ij}}{\sum_{i=1}^{m}\sum_{j=1}^{n}p_{ij}\sqrt{a_{ij}}}\right]\cdot {\left[1-\frac{1}{\sqrt{Z}}\right]}^{-1}\cdot \left(100\%\right)$$

where *p_ij_* is the perimeter of patch *ij* in terms of number of cell surfaces, *a_ij_* is the area of patch *ij* in terms of the number of cells, and *Z* is the total number of cells in the landscape.

### 2.4. Multi-Scales Analysis

In order to make clear the characteristics and differences of the distribution of land use types, this study assessed the percentage of land use types in the research area by county, elevation, and watershed scales. According to the survey of forest resources planning and natural physiognomy in the study areas, the relative elevation of the study areas was segmented into five sub-regions, which included I (0–100 m), II (100–250 m), III (250–500 m), IV (500–1000 m), and V (1000–2500 m). The current study analyzed the percentage of land use and the changes of landscape patterns from the five sub-regions, standardized the areas of land use in the three counties, and calculated the proportion of land use on average at different elevations, which better represented the characteristics of land use at specific elevations. The Soil and Water Assessment Tool (SWAT) and Digital Elevation Model (DEM) were used to divide the three research areas into several sub-basins. SY, ZS, and SC were divided in 35, 42, and 32 sub-basins, respectively. The percentage of different land use types in each sub-basin was calculated, and the correlation of different land use types at the watershed scale was analyzed.

## 3. Results

### 3.1. Landscape Patterns of Land Use Types at the County Scale

**Figure 3:**
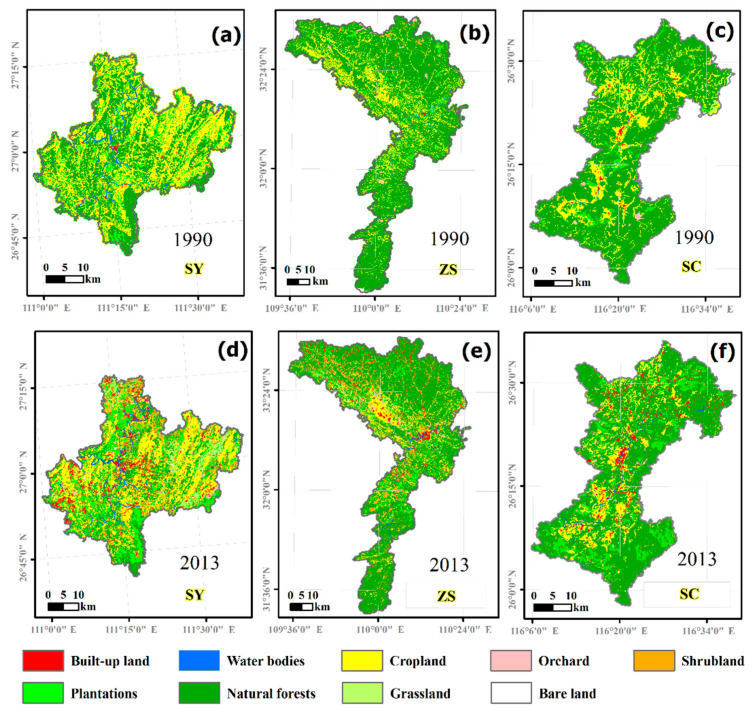
Distribution of land use types in the study area (**a**–**c**) in 1990, and (**d**–**f**) in 2013.

The main land use types of the three counties were NF and CL in 1990, which together accounted for more than 85% of the total land use types (Figure 3 and Table 3). From 1990 to 2013, the land use of the three counties changed significantly, among which NF, CL, PT, BUL, SL, and OC showed the same trend of change. Among them, the NF and CL in the three counties decreased significantly. The NF in SY, ZS, and SC decreased by 33.47%, 51.86%, and 34.75%, respectively, and the CL decreased by 43.02%, 14.82%, and 10.03%. PT increased by 67.02%, 8.17%, and 11.55%, BUL increased by 5.31, 18.40, and 4.00 times. In addition, SL and OC also showed an increasing trend in the study period.

From the landscape scale, the LPI, MPS, and IJI of the three counties all showed a decreasing trend during the study period. This suggests that the landscape pattern in all three counties had become more fragmented and heterogeneous. Among the three counties, SY had the largest decrease in LPI, which decreased from 11.18% to 5.45%, with a decrease of 51.25%, indicating that the SY big patches decreased the most obviously. The MPS of SC decreased from 19.52% to 15.79% and 19.11%, respectively, and the increase in patch fragmentation was sharp. The PAFRAC and COHESION of the three counties showed an increasing trend, indicating that the shapes of land use patches in the three counties had become more complex during the study period. The direct distance between the same type of patch became closer. The aggregation of the landscape pattern had increased (Table 4).

Although the changes in the landscape metrics of different land use types in the three counties were different, the change trend was basically the same from 1990 to 2013. During the study period, the PLAND, LPI, MPS, and COHESION of PL, BUL, GL, SL, and OC all increased, while PAFRAC and IIJ decreased. Among them, the change of BUL and SL were more obvious. The results showed that the landscape heterogeneity of PL, BUL, GL, SL, and OC decreased, and their spatial aggregation and connectivity increased. On the contrary, the landscape patterns of NF, CL, and WB showed the trend of fragmentation in the period (Table 5).

During the study period, MPS of NF in SY decreased from 6.31 ha in 1990 to 5.83 ha in 2013. Compared with ZS and SC, the landscape pattern of NF in SY was the most fragmented in both 1990 and 2013. The patch shape of NF in SY was also the simplest and the connectivity was the lowest. Compared with the other two counties, the landscape pattern of PL in SY was always the most fragmented. In 1990, the MPS of PT in SY was only 39.79% of SC. In 2013, the MPS of PL in SY was only 40.43% of SC. On the contrary, the PLAND, LPI, MPS, and COHESION of BUL and CL both were the largest in SY compared with ZS and SC. The MPS of CL in SY was 1.75 times of ZS and 1.91 times of SC. This shows that the landscape aggregation and connectivity of BUL and CL in the SY county were increased, while the forest land (NF and PT) was seriously fragmented (Table 5).

### 3.2. Landscape Patterns of Land Use Types along Altitude Gradients

**Figure 4:**
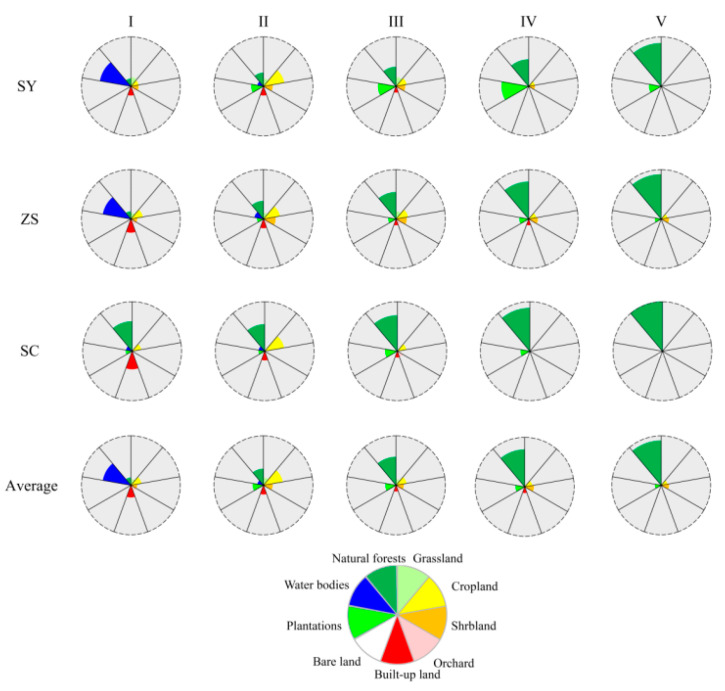
I (0–100 m), II (100–250 m), III (250–500 m), IV (500–1000 m), and V (1000–2500 m) represent five sub-regional divisions by elevation in Shaoyang (SY), Zhushan (ZS), and Shicheng (SC) counties in 2013. The flower diagrams illustrate the land use percentages of five sub-regions with different elevations by petal length. Each flower represents the percentage of land use types for one sub-region in SY, ZS, and SC.

The NF and CL accounted for more than half of the overall areas in II in 2013 (Figure 4). The proportion of NF and CL in II were 54.25% in SY, 55.91% in ZS, and 79.54% in SC. The PT were much larger in the II, III, and IV than in the other sub-regions (I and V). The NF increased from I (6.27%–55.77%) to V (84.02%–97.28%).

The WB was mainly concentrated in I (more than 50%) in the three counties. The GL and SL were evenly distributed. The BUL increased from I (16.72%) to II (8.45%), and decreased to V (0.18%) in the three counties (Figure 4). The CL increased from I (13.22%) to II (32.75%) and decreased to V (2.58%). The NF increased from I (6.43%) to V (87.58%). The PT were concentrated in II (12.34%), III (14.04%), and IV (10.76%), and accounted for 4.33% in the sum of I and V.

**Figure 5:**
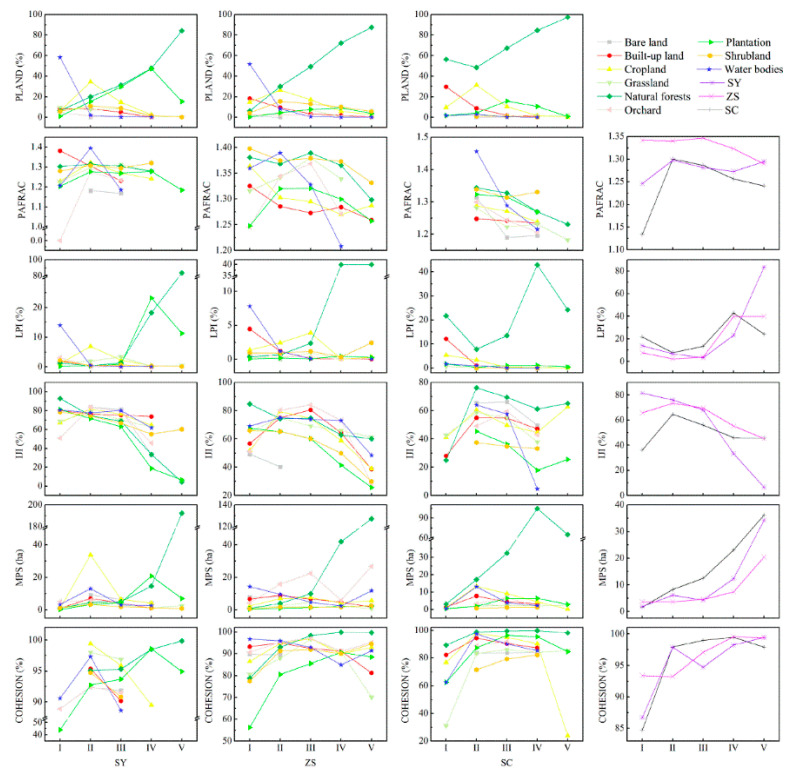
Variations and average trends of landscape indices along the altitude gradients in Shaoyang (SY), Zhushan (ZS), and Shicheng (SC) counties in 2013.

The LPI were the smallest and PAFRAC was the largest in II and III than in the other sub-regions (Figure 5). The MPS tended to decrease with increasing altitude in the three counties, in II and III, than the other sub-regions. The IJI increased from I to II, and decreased from II to III. The changes of COHESION basically showed a tendency to increase with altitude.

**Figure 6:**
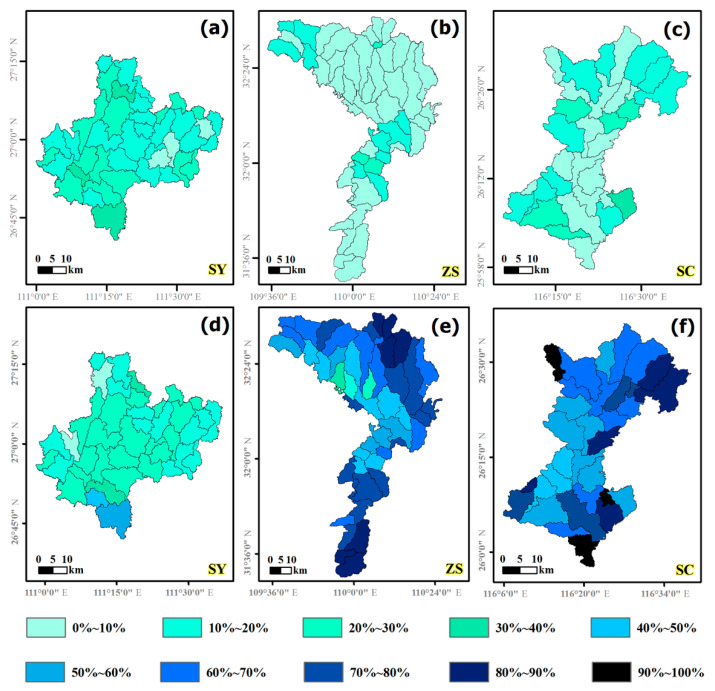
Percentage of plantations and natural forests in 2013 at the watershed scale (number of sub-basins, *n* = 35, 42, and 32 in SY, ZS, and SC). (**a**–**c**) Percentages of plantations in SY, ZS, and SC. (**d**–**f**) Percentages of natural forests in SY, ZS, and SC.

Along the altitude gradients, the PLAND of PT increased from I to IV, and then decreased to V in SY and ZS. The PT had the largest LPI value at 15.79% in III, and 11.29% in IV in SY, however, it was only <1% in ZS and <2% in SC, respectively. Overall, the IJI of PT decreased from I to V. The MPS of PT in I (0.23–0.54 ha) and II (1.05–3.3 ha) were the lowest compared with other land use types in the three counties. The MPS was bigger in NF than in PT, except for IV of SY. The COHESION increased from I to IV, and then decreased to V in PT, being lower than that in NF (Figure 6).

### 3.3. Landscape Patterns of Land Use Types at the Sub-Basin Level

**Figure 7:**
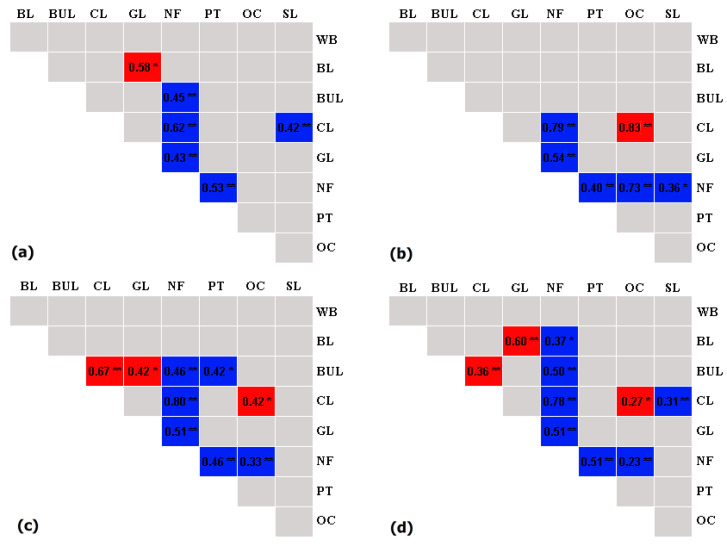
Pairwise relationships between areas of land use types at the watersheds scale in 2013. (**a**) The relationship of land use types in SY, (**b**) the relationship of land use types in ZS, (**c**) the relationship of land use types in SC, and (**d**) the relationship of land use types. Blue represents negative relationships and red represents positive relationships. White represents relationships with no established inter-relationship. The data in the frame represents the correlation coefficients between land use types. * Means that *p* less than 0.05; ** means that *p* less than 0.01. BL refers to bare land, BUL to built-up land, CL to cropland, GL to grassland, NF to natural forests, PT to plantations, OC to orchard, SL to shrubland, and WB to waterbodies.

According to the distribution of the land use types in the watersheds, the proportion of NF in the three counties was far more than the PT (Figure 7). In SY, PT accounted for 10–30% of the land area with a major fraction in the northwest and southern portions of the county, and NF accounted for 20–40%, with a major fraction in the central and southern portions of the county. In ZS, the area of PT typically accounted for 0–10%, while NF accounted for more than 40%. In SC, the proportion of PT accounted for 0–20% in the central region, and 10–20% in the surrounding area, while the NF typically accounted for more than 60%.

### 3.4. Correlations between Land Use Types

According to the correlation analysis of land use in different watersheds in 2013, the NF were negatively correlated with CL, PT, and GL (*p* < 0.01) in three counties (Figure 7). NF and BUL showed a significant negative correlation (*p* < 0.01) in SY and SC, but not in ZS. The OC and CL were significantly positively related in ZS (*p* < 0.01) and SC (*p* < 0.05). For all data combined in the three counties, there were negative correlations of NF with BL, BUL, CL, PT, GL, and OC, and a negative correlation between SL and CL, but positive correlations between CL and BUL, between GL and BUL, and between OC and CL (Figure 7).

## 4. Discussion

### 4.1. Characteristics of Forest Landscape Patterns

Our results clearly demonstrated that the landscape patterns of the study area were more fragmented in contrast to most counties of Southern and Northern China [34,35,36]. This is because the study areas were located in the middle reaches of the Yangtze River as the central parts of China where there was a longer developmental history, and the natural landscape was more strongly influenced by anthropogenic activities compared with most other areas in China [16,37,38]. This also implies that it is a harder task to improve the quality of plantation stands and to raise the level of ecosystem services across this region. In this context, there were two prominent features of forest landscape patterns as follows.

First, the forests (including natural forests and plantations) were the dominant one among the land use types identified in this study (Table 3), albeit with high fragmentation and heterogeneity (Table 5). Compared with the landscape patterns of the forests in Taishan Mountain, Qilian Mountain, and the middle Qinling Mountain, etc. across China, the forests in the study area were more fragmented [19,39,40,41,42]. Concurrently, the forest landscape of SY was also more fragmented than some of the other mountainous regions of the world [43,44]. This was revealed in recent years, although many plantations have been established and the forest coverage appears to be improved. People took for granted that a manmade ecological environment could be recovered, but there could be a tremendous gap between the landscape pattern and the original ecological environment [22,45].

Second, the PT were more fragmented and heterogeneous than the NF, which suggested that plantations were more strongly controlled in this region. This phenomenon was consistent with the landscape patterns of plantations in the Loess Plateau of China [38]. Fragmented forest landscapes lead to profound impacts on forest structures and compositions, causing changes in forest biodiversity and ecosystem functionality [23]. This can be confirmed by species diversity, soil quality, and fine root biomass, etc. in the soil profile of PT, which are relatively lower than NF [46,47].

### 4.2. Effects of Anthropogenic Disturbance on Land Use Types

From the perspective of socioeconomic development of the three counties, the urbanization level of SY, ZS, and SC existed at different levels. SY had the largest proportion of CL (29.36%) and BUL (7.44%), as well as a high GDP and population density compared with ZS and SC. The landscape pattern of SY was relatively fragmented and heterogeneous. The MPS of NF in SY was quite smaller than ZS and SC, which indicated that, presumably, anthropogenic activities, global warming, vegetation degradation, etc., strongly impacted the landscape patterns, and likely made the forests more fragmented.

Although various counties had different landscape characteristics, the change trends of the elevation related landscape patterns in the three counties were basically the same. With the increase in elevation, the number of large patches increased, the connectivity increased, and the patch shape gradually became simpler. Land use showed typical zonal distribution with elevation, and the proportion of NF steadily increased with altitude, however, the PT had a primary centralized distribution at 100–1000 m (>84.13%) in the three counties. The PAFRAC, MPS, and COHESION of PT also peaked from 100 to 1000 m. The homogeneity and connection of PT at the 100–1000 m elevation range were higher than at other altitudes, but still significantly lower than that of NF.

This indicates that there was still a gap of landscape patterns between the reconstructed forests and natural forests following anthropogenic destruction. It can also be proven that natural reserves and forest parks were always distributed at a relatively high altitude, where their species diversity and ecological aesthetic landscape value are relatively high [48]. The IJI of PT was the lowest of all the land use, indicating that PT were influenced by geographic distributed or artificial management, and their distribution was the most typical zonal distribution among all land use types.

### 4.3. Important Implications of the Results

Ecosystem services provided by forests are closely associated with both the total areas and distribution patterns [49]. For example, under the isolation effect of habitat patches, the migration and colonization of biological populations are limited [50,51]. The population of the habitat patches is smaller, and the gene exchange of the population is limited [52]. Landscape pattern changes can also be implicated in relation to the alteration of soil nutrients, hydrological cycles, and microclimates in the watershed [53]. Therefore, when implementing plantations, landscape patterns should be taken into account to avoid the negative effects brought about by fragmentation and habitat isolation.

Based on the geographical locations of three counties and the landscape patterns of forest vegetation, our results demonstrated the potential implications of natural resource conservation in this region where there has been a rapid socioeconomic development. First, it is essential to balance the economic development and natural protection for counties such as Shicheng and Zhushan. Our results revealed that there was a higher forest coverage (Figure 3 and Table 3), but a less developed economy in Shicheng and Zhushan counties [34,35,36]. However, the local people and governments have strong desires to develop the economy through the use of natural resources, where the simplest and fastest strategies are to cut forests to sell timber and to develop economic plantations, such as orange, bamboo, conifers, etc., to substitute protection forests for increasing the income of the local people. In order to mitigate such conflicts between economic development and environmental conservation in these areas, one feasible and efficient strategy is to implement an ecological compensation policy [5].

Second, our results clearly showed that the forest coverage was from 40% in SY to 80% in SC at the county scale, which was much higher than the mean value at the national level (21%), however, most of the forests were in remote and steep-sloped areas that were highly fragmented and uneven (Table 4 and Figure 5). This results in a reduction in the actual ecosystem services of forests, in particular, water and soil conservation, biodiversity, and recreation [46,47]. Thus, there are two silvicultural measures to be stressed in forest management for this region. One is to manage plantations in terms of nature-based orientation management philosophy such that plantation stands might be translated to high ecological benefit forest ecosystems [54,55]. This measure has been widely recognized in China [32,56]. Across the study areas, evergreen and deciduous broadleaf mixed forests are the zonal vegetation, with dominant tree species such as *Cinnamomum* spp., *Quercus* spp., *Schima* spp., and *Castanopsis* spp. (Figure 2d–f) [3,57]. Thus, with this nature-based management strategy, local native broadleaf tree species should be introduced in the artificial coniferous plantations to form the broadleaf-dominant forests in this area.

One is to increase forest coverage by afforestation in bare lands and to establish shelter forests around fruit gardens and villages, as well as along roads and rivers. Across this region, there are strong conflicts between economic development (e.g., fruit garden, oil-tea camellia plantations, tourisms, animal breeding, etc.) and environment protection (e.g., afforestation, protection zone, etc.), and land areas for afforestation are limited [58]. In this context, one feasible way is to establish native-species plantations as the shelter forests around fruit gardens which are necessary both for ensuring a high yield for fruit production and for increasing ecological services. Furthermore, establishing shelter-belt or landscape forests around villages and along roads and rivers are also favorable for local citizens and necessary for governments according to the national development strategy [59,60].

## 5. Conclusions

This paper analyzed the changes of land use types and landscape patterns at different scales in three typical counties in the middle reaches of the Yangtze River. The conclusions were as follows: (1) Compared with natural forests, plantations had smaller patches and a higher fragmentation degree; (2) the landscape patterns change of plantations were opposite to that of natural forests. The natural forests showed an increasing trend of fragmentation, while the plantations gathered from 1990 to 2013; (3) the areas of natural forests increased with the increase of altitude, the mean patch size increased and aggregation increased, while the areas of plantations reached the peak between 100 and 1000 m; (4) the areas of natural forests were negatively correlated with land use types such as bare land, built-up land, cropland, plantations, grassland, and orchard. These results indicated that the natural forests were gradually fragmented, and the plantations patches were characterized with fragmentation and unevenness under strong anthropogenic disturbances, with strong disagreements in regards to the near natural patterns and functions that were desired in this region. In order to solve these problems, the middle reaches of the Yangtze River region should take corresponding forest management measures to improve the landscape connectivity of plantations and natural forests, not only to enhance the areas of forests, but also to pay attention to the quality.

## Figures and Tables

**Table 1:** Land use types in the study area (based on the Yi and Chinese National Standard) [32,33].

Land Cover Types	Description
Grassland (GL)	Refers to growing herbaceous plants, including pasture or mainly pasture.
Cropland (CL)	Refers to land for planting crops, including cultivated land, new open wasteland, wheeled land, and crop fields so as to cultivate rice, lotus root, and other aquatic crops.
Shrubland (SL)	Short woodlands and shrubs.
Orchard (OC)	Refers to many years of intensive planting of woody and herbaceous crops for the intensive management of fruit, leaves, roots, stems, and juices.
Built-up land (BUL)	All types of manmade structures: Residential, industrial, agricultural commercial and services; transportation and utilities.
Bare land (BL)	The surface is rock or gravel, bare soils, sand, and bare stone.

**Table 2:** Description of landscape configuration metrics [10].

Structural Category	Landscape Metrics	Abbreviation	Description
Area/Density	Percentage of Landscape	PLAND	Measures the percentage of landscape
	Largest Patch Index	LPI	Area of the largest patch
	Mean Patch Area	MPS	The average mean surface of patches
Shape	Area-Weighted Mean Fractal Dimension Index	PAFRAC	Fractal dimension: Ratio of perimeter per unit area. Increases as patches become more irregular
Isolation and Interspersion	Interspersion Juxtaposition Index	IJI	Proximity of patches in each class. High values correspond to proportionate distribution of patch type adjacencies
Connectivity	Patch Cohesion Index	COHESION	Increases as the patches of the corresponding patch type become less connected

**Table 3:** The land use types and proportions in the study areas.

Land Use Types	SY				ZS				SC			
	1990		2013		1990		2013		1990		2013	
	Area (km^2^)	Percent (%)	Area (km^2^)	Percent (%)	Area (km^2^)	Percent (%)	Area (km^2^)	Percent (%)	Area (km^2^)	Percent (%)	Area (km^2^)	Percent (%)
GL	3.36	0.17	147.86	7.39	2.41	0.07	102.17	2.85	6.82	0.44	6.9	0.44
CL	885.45	44.25	589.08	29.44	775.85	21.62	373.48	10.41	324.92	20.73	212.01	13.53
SL	4.03	0.20	207.77	10.38	20.57	0.57	390.96	10.9	1.35	0.09	13.78	0.88
OC	6.04	0.30	27.81	1.39	35.98	1.00	36.78	1.03	10.49	0.67	20.74	1.32
BUL	23.34	1.17	147.28	7.36	5.02	0.14	97.39	2.71	10.27	0.66	51.34	3.28
BL	0.46	0.02	3.62	0.18	0.58	0.02	0.16	0.00	0.58	0.04	24.52	1.56
PT	227.32	11.36	379.67	18.97	56.91	1.59	275.15	7.67	55.76	3.56	188.91	12.05
NF	813.87	40.67	463.74	23.18	2669.73	74.41	2274.03	63.38	1152.36	73.52	1036.78	66.15
WB	37.12	1.86	34.18	1.71	20.77	0.58	37.7	1.05	4.85	0.31	12.43	0.79
Total	2001.01	100	2001.01	100	3587.81	100.00	3587.81	100	1567.4	100	1567.4	100

**Table 4:** Landscape indices of Shaoyang (SY), Zhushan (ZS), and Shicheng (SC) counties.

Counties	LPI (%)		MPS (ha)		PAFRAC		IJI (%)		COHESION (%)	
	1990	2013	1990	2013	1990	2013	1990	2013	1990	2013
SY	11.18	5.45	8.95	6.71	1.18	1.29	78.95	74.95	97.89	98.01
ZS	33.56	30.64	9.59	7.74	1.27	1.32	68.95	61.95	98.99	99.62
SC	55.26	52.4	19.52	15.79	1.2	1.26	69.4	61.4	98.87	99.74

**Table 5:** Landscape indices of land use types in Shaoyang (SY), Zhushan (ZS), and Shicheng (SC) counties.

Counties’ Land Use Types		PLAND (%)		LPI (%)		MPS (ha)		PAFRAC		IJI (%)		COHESION (%)	
		1990	2013	1990	2013	1990	2013	1990	2013	1990	2013	1990	2013
SY	NF	30.61	23.03	2.45	2.03	6.31	5.83	1.21	1.31	74.62	73.61	97.23	96.81
	PT	16.11	18.84	3.58	1.02	5.98	6.49	1.03	1.01	71.25	69.22	96.65	96.78
	BUL	5.12	7.34	0.32	0.41	6.41	8.61	1.21	1.3	78.25	77.16	94.23	95.3
	CL	35.98	29.71	6.21	5.45	34.12	33.32	1.36	1.32	79.25	81.72	99.56	99.38
	WB	2.95	1.72	1.06	1.07	16.21	15.19	1.36	1.35	77.9	78.5	98.92	98.81
	GL	3.21	7.45	1.63	1.65	1.23	5.26	1.27	1.29	77.98	78.38	97.68	98.06
	SL	5.12	10.34	0.36	0.38	1.48	3.28	1.32	1.31	74.45	73.73	93.62	94.71
	OC	0.79	1.39	0.15	0.22	5.98	7.35	1.25	1.28	83.98	84.08	93.98	94.04
	BL	0.11	0.18	0.01	0.02	1.08	3.41	1.15	1.14	86.54	85.94	94.32	93.49
ZS	NF	69.97	63.92	29.32	30.64	35.23	33	1.36	1.38	73.21	67.05	99.89	99.88
	PT	5.62	7.46	2.46	0.23	6.45	7.06	1.21	1.17	49.25	47.82	94.36	95.88
	BUL	1.31	2.71	0.09	0.11	6.32	8.13	1.23	1.3	78.21	77.19	92.15	93.94
	CL	12.02	10.41	2.32	1.93	12.78	8.7	1.34	1.31	70.21	68.91	97.69	96.97
	WB	2.32	1.06	0.62	0.35	25.65	21.5	1.38	1.43	76.23	77.28	98.56	98.01
	GL	2.1	2.86	0.98	0.41	1.02	4.83	1.31	1.37	64.2	66.43	96.52	96.61
	SL	6.13	10.55	0.75	0.61	1.42	2.04	1.34	1.37	51.86	53.63	92.98	93.16
	OC	0.52	1.03	0.09	0.11	5.79	7.63	1.26	1.29	79.65	79.53	95.32	96.99
	BL	0.01	0.01	0.01	0.01	3.2	5.48	1.14	1.15	62.13	57.92	93.25	92.42
SC	NF	68.9	66.35	51.89	52.4	63.12	53.91	1.34	1.31	74.87	74.4	99.87	99.93
	PT	7.52	11.98	3.22	0.77	15.03	16.05	1.27	1.20	43.21	36.75	97.21	97.95
	BUL	2.58	3.25	0.18	0.24	5.78	7.51	1.19	1.25	62.63	59.74	92.16	93.76
	CL	16.98	13.54	1.21	0.81	11.98	7.95	1.32	1.29	63.21	58.28	98.23	97.16
	WB	1.35	0.8	0.32	0.3	19.62	13.88	1.42	1.4	69.12	65.04	98.12	97.54
	GL	1.26	1.79	0.01	0.02	1.13	2.42	1.19	1.23	58.32	55.46	89.12	85.82
	SL	0.21	0.8	0.02	0.01	0.69	1.03	1.26	1.31	39.63	37.09	91.12	88.37
	OC	1.12	1.32	0.03	0.04	5.98	7.22	1.19	1.24	59.13	58.51	91.18	92.32
	BL	0.08	0.16	0.01	0.01	1.03	2.69	1.17	1.21	63.13	64.4	83.12	84.38

## Data Availability

Not applicable.

## Appendix A

**Table A1:** Counties, land use types, and landscape metrics abbreviations.

Types	Abbreviation	Content
Counties	SY	Shaoyang
	ZS	Zhushan
	SC	Shicheng
Land use types	GL	Grassland
	CL	Cropland
	SL	Shrubland
	OC	Orchard
	BUL	Built-up land
	BL	Bare land
	WB	Water bodies
	PT	Plantations
	NF	Natural forests
Landscape metrics	PAND	Percentage of Landscape
	LPI	Largest Patch Index
	MPS	Mean Patch Area
	PAFRAC	Area-Weighted Mean Fractal Dimension Index
	IJI	Interspersion Juxtaposition Index
	COHESION	Patch Cohesion Index
